# Gender Disparity in Citations in High-Impact Journal Articles

**DOI:** 10.1001/jamanetworkopen.2021.14509

**Published:** 2021-07-02

**Authors:** Paula Chatterjee, Rachel M. Werner

**Affiliations:** 1 Division of General Internal Medicine, Department of Medicine, Perelman School of Medicine at the University of Pennsylvania, Philadelphia; 2 The Leonard Davis Institute of Health Economics, University of Pennsylvania, Philadelphia; 3 Penn Presbyterian Hospital, Philadelphia, Pennsylvania; 4 The Corporal Michael J. Crescenz Veterans Affairs Medical Center, Philadelphia, Pennsylvania

## Abstract

**Importance:**

Women are less likely to be promoted and hold leadership positions in academic medicine. How often academic articles are cited is a key measure of scholarly impact and frequently assessed for professional advancement; however, it is unknown whether peer-reviewed articles written by men and women are cited differently.

**Objective:**

To evaluate whether academic articles from high-impact medical journals written by men and women are cited differently.

**Design, Setting, and Participants:**

In this cross-sectional study of all original research and commentary articles from 5 high-impact medical journals (*Annals of Internal Medicine, British Medical Journal, JAMA, JAMA Internal Medicine,* and *The New England Journal of Medicine*) from 2015 to 2018, the gender of the primary and senior authors of each article were identified using an online database, and the number of times each article has been cited was identified using Web of Science. The number of citations by primary and senior author gender were then compared. Data were analyzed from July 2020 to April 2021.

**Exposures:**

Primary and senior authors’ genders.

**Main Outcomes and Measures:**

Number of citations per article.

**Results:**

Among 5554 articles, women wrote 1975 (35.6%) as primary author and 1273 of 4940 (25.8%) as senior author. Original research articles written by women as primary authors had fewer median (interquartile range) citations than articles written by men as primary authors (36 [17-82] citations vs 54 [22-141] citations; *P* < .001) and senior authors (37 [17-93] citations vs 51 [20-128] citations; *P* < .001). Articles written by women as both primary and senior authors had approximately half as many median (interquartile range) citations as those authored by men as both primary and senior authors (33 [15-68] citations vs 59 [23-149] citations; *P* < .001). Differences in citations remained in each year of the study and were less pronounced among commentary articles.

**Conclusions and Relevance:**

In this study, articles written by women in high-impact medical journals had fewer citations than those written by men, particularly when women wrote together as primary and senior authors. These differences may have important consequences for the professional success of women and achieving gender equity in academic medicine.

## Key Points

**Question:**

Are academic articles written by men and women in high-impact medical journals cited differently?

**Findings:**

In this cross-sectional study of 5554 articles, those written by women primary or senior authors had fewer citations than those written by men primary or senior authors. Articles written by women as both primary and senior authors had approximately half the number of citations as those authored by men as both primary and senior authors.

**Meaning:**

These findings suggest that gender-based differences in article citations may be a key contributor to disparities in the advancement and promotion of women in academic medicine.

## Teaser

This cross-sectional study evaluates whether academic articles in high-impact medical journals written by men and women are cited differently.

## Introduction

Women in academic medicine face myriad professional challenges. While women are increasingly entering the field,^1^ they are less likely to be recognized as experts and leaders, with fewer women speaking at national medical conferences or grand rounds,^2,3^ receiving prestigious awards,^4^ being promoted to full professorships,^5^ or holding leadership roles.^6^ This observed gender gap in academic achievements may be attributable, in part, to lower levels of research productivity. Women are less likely to author original research, guest editorials, and commentaries in major journals,^7,8,9^ particularly as first author,^10^ which may be the result of numerous barriers women face in academic medicine. It is also possible that when women are successful in their research, they receive less recognition for it.

Recognition and amplification of academic achievements are important factors for success, including professional advancement and appointment to leadership positions. If women are less likely to be recognized for their contributions and seen as experts in their fields, it could inhibit their advancement and promotion. Prior research has shown that women in academic medicine are less likely to be amplified on social media platforms,^11^ which are increasingly used for research dissemination. However, little is known about whether scholarly impact and academic influence differ between men and women.

The number of citations of peer-reviewed articles is an important indicator of scholarly impact. It is commonly used as a metric for academic recognition, influence, and acceptance by scientific communities as well as in professional evaluations and promotion.^12,13^ However, it is unknown whether articles written by men and women in academic medicine are cited differently. In this study, we examined whether there is a gender gap in the number of citations of peer-reviewed publications at 5 high-impact academic medical journals, measuring the number of citations and comparing them by the genders of primary and senior authors.

## Methods

### Data

We collected data on articles published in 5 leading academic medical journals between 2015 and 2018: *Annals of Internal Medicine, British Medical Journal, JAMA, JAMA Internal Medicine,* and *The New England Journal of Medicine (NEJM)*. Similar to prior work,^7^ we chose these 5 journals using a combination of impact factor evaluation and consensus-based discussion with academic medical faculty about the long-term professional impact of publication in these journals. Within each journal, we included all peer-reviewed original research articles (including full-length articles, brief reports, and research letters) and commentaries (including viewpoints and perspectives). We excluded narrative essays, due to the low frequency of citation, as well as clinical guidelines, which are typically written by expert consortia or organizations as opposed to individual authors. We also excluded editorials, systematic reviews, and narrative reviews because the primary focus of this study was citations of original research investigations. For a list of article subtypes included from each journal, see the eTable in the Supplement. Per the Common Rule and owing to the use of publicly available data, this study was exempt from institutional review board approval and the requirement for informed consent. This study abides by the Strengthening the Reporting of Observational Studies in Epidemiology (STROBE) reporting guideline for cross-sectional studies.

For each article, we collected the names of primary (or first) and senior (or last) authors and the date of publication. To determine author gender, we used Genderize, an online database that has been used in prior work examining gender disparities in authorship of academic articles.^14,15,16^ This database assigns genders to names based on how frequently a name occurs in public social media profiles where the gender of the user can be verified.^17^ For each name it successfully classifies, the tool also estimates the probability of correct gender classification. For names that could not be classified by the tool or for which the probability of correct gender classification was less than 0.9, we performed a manual internet-based search of authors’ names and affiliations to classify gender.

For each published article, we obtained the number of times the article had been cited to date through the Web of Science. All data collection was performed between May 11 and July 4, 2020. Using the total number of citations, date of article publication, and the date of data collection, we also calculated the median number of citations per year since publication.

### Statistical Analysis

Data from 5665 articles were collected, from which we excluded 52 articles (0.9%) for which authorship was ascribed to a research consortium and 59 articles (1.0%) for which gender could not be determined. Of the remaining 5554 articles, there were 614 articles (11.1%) with only 1 author. These articles were excluded from analyses of senior authorship.

First, we summarized the articles included in the sample by the genders of primary and senior authors, year of article publication, journal, and article type. Because of the right-skewed distribution of citations introduced by positive outliers, we then compared differences in the median (interquartile range [IQR]) number of citations by gender and by year. Finally, we examined differences in citation counts between different gender combinations of primary and senior author pairs. We performed nonparametric equality of medians tests to compare the number of citations between paired groups and Kruskal-Wallis tests between multiple groups. We applied 1-tailed tests with statistical significance determined at a threshold of *P* < .025. All analyses were performed separately for original research articles and commentaries and completed using Stata version 15 (StataCorp).

## Results

The sample included 5554 articles, of which 1975 (35.6%) had women as primary authors (Table 1). Of 4940 articles with more than 1 author, 1273 (25.8%) had women as senior author. There were more original research articles than commentaries (3354 [60.4%] vs 2200 [39.6%]), and the most articles were published by *JAMA* (1644 [29.6%]) and *NEJM* (1605 [28.9%]) during the study period.

**Table 1.** Sample Characteristics

Characteristic	Articles, No. (%) (N = 5554)
Primary authorship	
Woman	1975 (35.6)
Man	3579 (64.4)
Senior authorship^a^	
Woman	1273 (25.8)
Man	3667 (74.2)
Year	
2015	1449 (26.1)
2016	1339 (24.1)
2017	1384 (24.9)
2018	1382 (24.9)
Journal	
*Annals of Internal Medicine*	526 (9.5)
*BMJ*	937 (16.9)
*JAMA*	1644 (29.6)
*JAMA Internal Medicine*	842 (15.2)
*NEJM*	1605 (28.9)
Article type	
Original research	3354 (60.4)
Commentary	2200 (39.6)

Abbreviations: *BMJ*, *British Medical Journal*; *NEJM*, *The New England Journal of Medicine*.

^a^ A total of 614 articles had only 1 author and no senior author.

Original research articles with women as primary authors were cited fewer median (IQR) times than those with men as primary authors (36 [17-82] citations vs 54 [22-141] citations; *P* < .001) (Table 2). This pattern was consistent across each year included in the study period. When comparing the number of citations per year since publication, articles by women as primary authors had fewer median (IQR) citations over the study period (11.4 [5.4-23.4] citations vs 16.2 [7.0-40.4] citations; *P* < .001) and in each study year. Articles with women as senior authors were also cited fewer times than articles with men as senior authors overall (median [IQR], 37 [17-93] citations vs 51 [20-128] citations; *P* < .001) and per year since publication (median [IQR], 12 [5-25] citations vs 15 [7-37] citations; *P* < .001), although the magnitude of the difference in number of citations by gender was smaller for senior authors than for primary authors.

**Table 2.** Number of Citations for Original Research Articles Over Time, Stratified by Primary and Senior Author Gender

Year	Citations by primary author gender, median (IQR), No.		*P* value^a^	Citations by senior author gender, median (IQR), No.		*P* value^a^
	Woman	Man		Woman	Man	
Total citations						
Overall	36 (17-82)	54 (22-141)	<.001	37 (17-93)	51 (20-128)	<.001
2015	64 (27-140)	82 (34-198)	.02	67 (26-136)	79 (35-188)	.16
2016	45 (21-99)	69 (29-173)	.001	45 (21-115)	64 (28-149)	.05
2017	32 (14-66)	49 (3-130)	<.001	33 (15-63)	44 (19-112)	.01
2018	21 (12-41)	29 (12-69)	.03	21 (9-38)	28 (13-61)	.002
Citations per year						
Overall	11 (5-23)	16 (7-40)	<.001	12 (5-25)	15 (7-37)	<.001
2015	13 (5-28)	16 (7-39)	.02	13 (5-28)	16 (7-38)	.16
2016	12 (5-25)	18 (8-43)	.001	12 (5-30)	17 (8-39)	.05
2017	11 (5-21)	16 (7-45)	<.001	11 (5-23)	15 (7-37)	.03
2018	11 (6-21)	15 (6-35)	.01	11 (5-20)	15 (7-31)	.01

Abbreviation: IQR, interquartile range.

^a^ *P* values correspond to 1-tailed nonparametric equality of medians tests.

Original research articles with women as both primary and senior authors were cited the fewest times, with a median (IQR) of 33 (15-68) citations (Table 3), whereas articles authored by men as both primary and senior authors were cited most (median [IQR], 59 [23-149] citations). Articles with women as primary authors and men as senior authors had fewer citations than articles with men as primary authors and women as senior authors (median [IQR], 39 [17-89] citations vs 42 [19-119] citations). Comparisons across the 4 pairs of primary/senior author genders were statistically significant (*P* < .001).

**Table 3.** Total Number of Citations for Original Research Articles by Primary and Senior Authorship Gender Pairs

Author pair	Citations, median (IQR), No.	*P* value^a^
Woman primary and senior	33 (15-68)	<.001
Woman primary and man senior	38.5 (17-89)	
Man primary and woman senior	42 (19-119)	
Man primary and senior	59 (23-149)	

Abbreviation: IQR, interquartile range.

^a^ *P* value corresponds to 1-tailed Kruskal-Wallis test.

Finally, when we examined citations among commentary articles, differences by gender were attenuated. The difference in citations of articles where women were primary authors compared with those authored by men were not statistically significant (median [IQR], 8 [3-19] citations vs 10 [4-22] citations; *P* = .16) (Table 4).

**Table 4.** Number of Citations and Number of Citations per Year of Commentaries, Stratified by Primary and Senior Author Gender

Year	Citations by primary author gender, median (IQR), No.		*P* value^a^	Citations by senior author gender, median (IQR), No.		*P* value^a^
	Woman	Man		Woman	Man	
Total citations						
Overall	8 (3-19)	10 (4-22)	.16	9 (3-22)	11 (4-23)	.18
2015	12 (4-29)	13 (6-32)	.46	16 (6-30)	15 (6-34)	.75
2016	13 (5-25)	13 (4-25)	.82	19 (7-36)	13 (6-26)	.05
2017	8 (3-18)	8 (4-16)	.77	8 (3-16)	10 (5-19)	.01
2018	7 (2-13)	5 (2-12)	.03	5 (2-12)	7 (3-14)	.07
Citations per year						
Overall	3 (1-6)	3 (1-6)	.62	3 (1-7)	3 (2-7)	.14
2015	2 (1-6)	3 (1-6)	.51	3 (1-6)	3 (1-7)	.88
2016	3 (1-6)	3 (1-6)	.82	5 (2-10)	4 (2-7)	.05
2017	3 (1-6)	3 (1-6)	.43	2 (1-5)	3 (2-7)	.02
2018	3 (1-7)	3 (1-6)	.53	3 (1-6)	3 (1-7)	.09
Citations by senior author gender						
Woman	9 (4-21)	9 (3-23)	.17	NA	NA	NA
Man	11 (4.5-23)	11 (4-24)		NA	NA	NA

Abbreviations: IQR, interquartile range; NA, not applicable.

^a^ *P* values correspond to 1-tailed nonparametric equality of medians tests for total citations and number of citations per year (overall and year specific) and to 1-tailed Kruskal-Wallis tests for primary author–senior author comparisons.

## Discussion

In academic medicine, scholarly recognition and impact are necessary for promotion and professional advancement. The number of times that publications have been cited is a commonly used metric to quantify this scholarly impact.^12,13^ In this study of 5 high-impact medical journals, we found that original research articles written by women as primary authors had fewer citations than those written by men. These differences were consistent over time. Furthermore, women who coauthored with other women as senior authors had the fewest median citations, while men who coauthored with other men as senior authors had the most citations.

This work builds on prior literature documenting lower levels of visibility and amplification for women in academic medicine compared with men. Women are less likely to be quoted in the lay press as content experts,^18,19^ less frequently invited to speak at grand rounds or international conferences,^2,20^ and less likely to be referred to using their professional titles in academic contexts,^20^ all of which can contribute to well-established gender disparities in academic rank and leadership positions. While gender disparities in authorship have improved over time,^7,10^ our study confirms that women are still much less likely to be primary authors of articles published in high-impact medical journals. Women are even less likely to be senior authors, suggesting that the pool of women eligible for these positions remains small.

Our finding of fewer citations of articles authored by women is likely the result of multiple factors. Women have smaller professional networks, smaller audiences, and narrower reach on virtual platforms, which are emerging as a key tool for research dissemination.^11^ Women are less likely to be amplified on social media, where dissemination of research may be associated with higher citation counts.^21,22^ It is possible that women are more likely to author articles on topics that have smaller audiences and are less frequently cited. Some may also wonder whether articles by women are of lower caliber and, thus, less likely to be cited. However, by restricting this study to top-tier medical journals, we limited the likely effect of these factors. Some of the journals included in this study focus on the field of internal medicine, which typically has a higher proportion of women represented than other clinical specialties.^23^ As a result, our findings may underestimate differences observed in other fields of medicine in which there is less parity in gender distribution. Importantly, our findings are consistent with work from fields outside of academic medicine, which have shown that articles written by women have fewer citations than those written by men.^24,25^

Mitigating these disparities will be challenging. First, there is no supervisory bibliometric body to track citation disparities over time. Second, ensuring adequate representation among citations in peer-reviewed literature is difficult. Third, in part because of the largely invisible nature of this disparity, there are no consequences for its perpetuation. Nonetheless, mitigating these disparities is important. Citations continue to hold great importance in academic contexts. A researcher’s h index, defined as a composite measure of the number of academic articles and the citations of those articles, is increasingly reported by research databases, such as Google Scholar, Web of Knowledge, and Scopus. Some have advocated for the use of the h index in promotion and hiring decisions, while others have considered using it to define benchmarks for research productivity.^26,27,28^

Despite these challenges, there are several ways to move forward. Articles published in open-access journals tend to receive more citations,^29,30^ and the proliferation of these journals may narrow citation differences if women publish in them at growing rates. Journals could begin to measure diversity of representation among citations in their published articles and track their gender distribution for high-profile, invited commentaries. Journals and academic public relations offices could also ensure equal promotion of new research findings for all authors. Finally, scholarly impact is only one part of academic promotion and selection for leadership positions. Women continue to face barriers along other dimensions of advancement. Investing more in formal and peer mentoring programs, providing incentives for the sponsorship and promotion of women and others underrepresented in academic medicine, and ongoing efforts to reduce the consequences of implicit bias remain paramount.^31^

In all likelihood, our findings of gender disparities in citations represent the tip of the iceberg, and gender disparities are just one way in which inequities in academic medicine should be examined. Prior work has also shown significant inequities across race and ethnicity in academic medicine,^32,33,34^ and further work is needed to examine differences in scholarly recognition by these factors. Women from minority ethnic and racial groups may face particularly formidable barriers to professional advancement that are introduced by intersecting biases. Addressing the sociodemographic imbalances in academic medicine will require a multifaceted and intentional approach.

We conducted a separate citation analysis of the articles cited in this manuscript using the gender-identifying tool. Among 34 cited articles, we excluded 1 article written by an organization. Of the remaining 33 cited articles, 16 (48.5%) were written by women as first authors, while 17 (51.5%) were written by men. Given that between 30% and 40% of women serve as primary authors of high-impact articles, we consider our percentage of women authors cited to be higher than what would be expected from the distribution.

### Limitations

Our study has limitations. First, we only examined a subset of academic journals, and our findings may not be generalizable to all journals in academic medicine. The sample of journals in this study may overrepresent the field of internal medicine; however, as described previously, this would suggest that the disparities observed are an underestimation compared with other fields of medicine, in which the gender distribution is less equal. Second, we assigned gender using an external database, which may have resulted in misclassification. However, genders that were assigned with a low probability of accuracy were manually evaluated. Third, last authorship may not perfectly match senior authorship. Fourth, we focused on dichotomized gender categories and were unable to assess citation differences among authors with nondichotomous gender affiliations.

## Conclusions

In this study, articles written by women in high-impact medical journals had fewer citations than those written by men, particularly when women wrote together as primary and senior authors. This research sheds important and new light on the problem of gender equity in academic medicine. Women are not only less likely to be published as primary or senior authors in high-impact medical journals, but when they are, their publications are cited less frequently by their peers. These findings suggest that some of the observed gender disparities in academic medicine will not be solved by additional training and hiring of women. Rather, we must also focus on ensuring that women in academic medicine have a level playing field that equally values and promotes their successes.

## Notes

Supplement. **eTable. **Classifications of Article Subtypes by Journal
