# Errors in Table 2, Table 3, and Table 4

**DOI:** 10.1001/jamanetworkopen.2021.27739

**Published:** 2021-08-26

In the Original Investigation titled “Gender Disparity in Citations in High-Impact Journal Articles,”^1^ published July 2, 2021, there were rounding errors in the Results, Table 2, Table 3, and Table 4. In Table 2, citations by men as first authors in 2017 should have read 49 (21-130), citations per year overall by men should have read 15 (7-36), citations per year overall by men as senior authors in 2016 should have read 17 (7-39), and citations per year overall by men as senior authors in 2017 should have read 15 (6-37). In Table 3, citations by women as primary authors and men as senior authors should have read 39 (17-89). In Table 4, citations per year overall by men as senior authors should have read 3 (1-7), citations per year by men as senior authors in 2016 should have read 4 (1-7), and citations by women as primary authors with men as senior authors should have read 11 (5-23). This article has been corrected.^1^
