# Supplementary material for: Gender Disparity in Citations in High-Impact Journal Articles
Source: JAMA Netw Open. 2021 Jul 2;4(7):e2114509. doi: 10.1001/jamanetworkopen.2021.14509 (PMC8254129; doi:10.1001/jamanetworkopen.2021.14509)
Supplement: Supplement. — eTable. Classifications of Article Subtypes by Journal [file jamanetwopen-e2114509-s001.pdf]

Supplemental Online Content

Chatterjee P, Werner RM. Gender disparity in citations in high-impact journal articles. *JAMA Netw Open.* 2021;4(7):e2114509. doi:10.1001/jamanetworkopen.2021.14509

eTable. Classifications of Article Subtypes by Journal

This supplemental material has been provided by the authors to give readers additional information about their work.

© 2021 Chatterjee P et al. *JAMA Network Open.*

eTable. Classifications of Article Subtypes by Journal

Journal	Original Research Articles	Commentaries
Annals of Internal Medicine	Original Research	Ideas and Opinions
British Medical Journal	Research	Analysis
JAMA	Original Investigation, Preliminary Communication, Research Letter	Viewpoint
JAMA Internal Medicine	Original Investigation, Research Letter	Viewpoint
The New England Journal of Medicine	Original Article, Special Article	Perspective

© 2021 Chatterjee P et al. *JAMA Network Open.*
